# Stress-related cellular pathophysiology as a crosstalk risk factor for neurocognitive and psychiatric disorders

**DOI:** 10.1186/s12868-023-00831-2

**Published:** 2023-12-12

**Authors:** Iryna S. Palamarchuk, George M. Slavich, Tracy Vaillancourt, Tarek K. Rajji

**Affiliations:** 1 Centre for Addiction and Mental Health, 1001 Queen Street West, Toronto, ON M6J1H4 Canada; 2 Department of Psychiatry, Temerty Faculty of Medicine, University of Toronto, Toronto, ON Canada; 3 Sunnybrook Health Sciences Centre, Division of Neurology, Toronto, ON Canada; 4 Temerty Faculty of Medicine, Toronto Dementia Research Alliance, University of Toronto, Toronto, ON Canada; 5 Department of Psychiatry and Biobehavioral Sciences, University of California, Los Angeles, Los Angeles, CA USA; 6 Counselling Psychology, Faculty of Education, University of Ottawa, Ottawa, ON Canada; 7 School of Psychology, Faculty of Social Sciences, University of Ottawa, Ottawa, ON Canada

**Keywords:** Anxiety, Cognition, Depression, Epigenetics, Memory, Neurodegeneration, Kinase, Psychological stress, Synaptic activity

## Abstract

In this narrative review, we examine biological processes linking psychological stress and cognition, with a focus on how psychological stress can activate multiple neurobiological mechanisms that drive cognitive decline and behavioral change. First, we describe the general neurobiology of the stress response to define neurocognitive stress reactivity. Second, we review aspects of epigenetic regulation, synaptic transmission, sex hormones, photoperiodic plasticity, and psychoneuroimmunological processes that can contribute to cognitive decline and neuropsychiatric conditions. Third, we explain mechanistic processes linking the stress response and neuropathology. Fourth, we discuss molecular nuances such as an interplay between kinases and proteins, as well as differential role of sex hormones, that can increase vulnerability to cognitive and emotional dysregulation following stress. Finally, we explicate several testable hypotheses for stress, neurocognitive, and neuropsychiatric research. Together, this work highlights how stress processes alter neurophysiology on multiple levels to increase individuals’ risk for neurocognitive and psychiatric disorders, and points toward novel therapeutic targets for mitigating these effects. The resulting models can thus advance dementia and mental health research, and translational neuroscience, with an eye toward clinical application in cognitive and behavioral neurology, and psychiatry.

## Background

Stress is a physiological response that engages the hypothalamic–pituitary–adrenal (HPA) axis and locus coeruleus-norepinephrine (LC-NE) system to address salient stimuli (i.e., stressors) that are either potential or actual threats to our physical and/or psychological well-being. Psychological stressors trigger negative effects on mental health known as psychological stress [e.g., 1, 2]. The magnitude and prevalence of psychological stress effects are well documented. For example, in the United States, work-related stress has an economic impact of up to $190 billion annually [3, 4], and the results of the 2022 survey performed for the American Psychological Association showed that stress is altering all aspects of life [5]. Accordingly, the World Health Organization highlighted the global importance of interventions for stress management and well-being programmes in its Comprehensive Mental Health Action Plan 2013–2030 [6].

Substantial research indicates that psychological stress, which can involve extreme and/or chronic experiences of aversive feelings, can evoke neural responses associated with pain perception [e.g., 7, 8], mentalizing, psychiatric disorders, and cognitive deteriorations [e.g., 1, 2, 9, 10]. Stress is also a risk factor for both acute and progressive cerebral pathologies such as stroke and neurodegenerative disease [e.g., 9, 11–14], which reciprocal interplay to accelerate cognitive manifestation of dementia [e.g., 15, 16]. Neurodegenerative diseases and psychiatric disorders are often highly comorbid [e.g., 17–19].

To better understand stress-induced neural dynamics implicated in cognitive impairment, as well as the etiopathogenetic mechanisms that can be shared by stress-related neurocognitive diseases and psychiatric disorders, we present a comprehensive narrative review of their interconnected cellular processes. The conceptual framework for this review is centered on the idea that psychological stress can alter the brain on multiple levels (e.g., epigenetics, neurotransmitters) that are relevant for affect, behavior, and cognition. First, we briefly revisit the general neurobiology of the stress response to define neurocognitive stress reactivity and the mechanisms of habituation and sensitization. Next, we review the main mechanisms shared by stress responses, cognitive processing, and neuropsychiatric alteration on the level of epigenetic regulation, synaptic transmission, sex hormone co-signalling, photoperiodic plasticity, and psychoneuroimmunology. The review is structured as an array of the sections on each level, as we describe how stress can contribute to cognitive decline and behavioral alteration. We show mechanistic parallelisms between stress response and neuropathology with the aim of proposing new experimental directions and specific scientific hypotheses that should be tested. Ultimately, understanding links between stress, cognitive impairment, and psychiatric disorders will enable researchers to generate testable hypotheses that can in turn lead to novel insights and potentially to new therapeutic targets.

## Neuropsychobiology of the stress responses

*Summary of the concepts*: Stress is a reaction to a stressor in attempt to protect and maintain physiological stability through the process of allostasis that helps adaptation. However, exposure to chronic or severe stress can cause habituation and sensitization associated with harmful effects of altered allostasis.

*Takeaway*: Stress responses are functions of allostatic status and cognitive appraisal.

### The concept of allostasis

Exposure to psychological stressors evokes neural responses directed to protect and maintain physiological stability (i.e., homeostasis) through a process known as allostasis [e.g., 20–22]. Allostasis is determined by the synergetic activities of the HPA axis (“neuroendocrine limb”) and the LC-NE system (“cognitive limb”) [e.g., 1, 9, 23–26]. Allostasis is initiated with the release of corticotropin-releasing hormone (CRH) from the hypothalamus and urocortin from the brainstem, which together form the corticotropin-releasing factor (CRF) family in humans. CRH release stimulates secretion of adrenocorticotropic hormone (ACTH) from the adenohypophysis, which, in turn, stimulates the adrenal cortex to produce cortisol as a stress hormone [e.g., 27–30]. The cortisol response to stress follows a triphasic pattern during a successful adaptation to stressors [e.g., 21, 22, 31]. First, there is an alarm reaction, then resistance, followed by resolution of the cortisol response due to negative feedback-inhibition over the HPA activity that results in decreased sensitivity to ACTH, and, in turn, suppression of cortisol production. This physiological pattern of cortisol response is moderated by sensitivity of the glucocorticoid receptors (GR).

However, when stress-induced neuroendocrine responses are ongoing or elevated, allostatic load can turn into allostatic overload to which there is a cost of poor health outcomes, including psychiatric disorders [e.g., 32]. The allostatic load can result from exposure to a chronic or repeated stressor (e.g., interpersonal stress), cumulative challenges (e.g., work-related stress and low socioeconomic status) or prior adverse experiences (e.g., maltreatment in childhood). That is why in patients, the same clinical diagnosis does not imply the same underlying health condition. Assessments of allostatic load, such as the evaluation of biopsychosocial determinants of health, may help with advancing personalised clinical interventions [e.g., 33].

Severe or chronic stress can result in allostatic failure defined as a stress response that surpasses the essential needs and, in turn, results in harmful effects (i.e., maladaptation) [e.g., 9, 10, 21, 24, 25, 31–35]. Importantly, stress severity—and, hence, the level of adaptation—is principally linked to a person’s cognitive appraisal of the situation (i.e., how the stressor is perceived; for details, please see our recent work [1]). This means that neurobiological responses to a psychological stressor are largely modulated by neuropsychological feedback, with an individual’s cognitive and behavioral changes in a response to a psychological stressor being called neurocognitive stress reactivity [1].

The neurocognitive responses play a crucial role in early-life stress/adverse childhood experiences. Specifically, the neurodevelopmental aspects increase brain vulnerability to cognitive and emotional dysregulation, which is a risk for childhood stress psychopathology (for details, see [2]). The neurocognitive impact of stress reactivity is also prominent in various neuropsychiatric conditions. For example, when coping with stress, individuals with borderline personality disorder or post-traumatic stress disorder (PTSD) can present with reduced pain perception related to dissociation which, in turn, can be linked to self-harm and suicide attempts [e.g., 36–38; see also 39]. Accordingly, the assessment of somatosensory function can serve as a clinical intervention tool for screening psychologically vulnerable populations whose stress-coping mechanisms may increase their risk for suicide attempts [e.g., 40, 41].

### Habituation and sensitization in response to stress

At the neuroendocrine level, maladaptive stress responses depend in part on an individual’s sensitivity to ACTH and the type of stressor experienced. Specifically, the stressor can be novel (i.e., acute exposure to a new aversive stimulus), homotypic (i.e., chronic/repeated exposure to the same/similar aversive stimuli, e.g., chronic bullying), or heterotypic (i.e., exposure to different aversive stimuli, e.g., financial or psychosocial challenges following caregiver burden). Sensitivity to ACTH can be: (a) increased for a novel stressor, (b) initially increased and then decreased during chronic exposure to a homotypic stressor, or (c) diminished in response to a homotypic stressor [42–44]. In addition, exposure to homotypic stressors can lead to habituation of the HPA axis response (as can be seen in decreased glucocorticoid response) and enhance post-stress recovery of the HPA axis (i.e., post-stress return to baseline). Yet, prior exposure to homotypic stressors can cause sensitization of the HPA axis response (as can be seen in increased glucocorticoid response) and worsen the recovery of the HPA axis following exposure to a novel stressor [e.g., 34, 45–47]. The outcomes of maladaptive stress-induced hyperactivity of the HPA axis seen in prolonged hypercortisolemia and/or altered GR function are related to depression and anxiety [e.g., 9, 48, 49]. Additionally, behavioral sensitization appears to persist longer than the HPA axis sensitization and is often a latent phenomenon revealed by a novel stressor [e.g., 47, also see 50]. In other words, stress adaptation declines in the context of chronic stress, which reduces a person’s ability to recover after a novel stressor exposure and can increase their susceptibility to mood disorders and other stress-related health problems [e.g., 1, 2].

At the same time, stress resilience is associated with CRF-mediated regulation of cognitive and behavioral activity [9, 21, 27–29, 31, 44, 51–55]. Amygdalar CRF signalling has anxiogenic-like effects [e.g., 9, 29] and mediates defensive responses such as increased vigilance, ability to discriminate salient stimuli, and active escape [e.g., 56]. Research has shown that stress-induced increases in the expression of amygdalar CRF-1 receptors are associated with deficient hippocampal-dependent memory and learning, as demonstrated in the impaired visual discrimination task [e.g., 29, 54, 55; see also 23], whereas hippocampal CRF signalling can promote synaptic remodelling that is potentially supportive of memory consolidation in acute stress [e.g., 57]. To complicate matters, chronic stress also promotes connectivity between the amygdala and striatum. The consequences can be seen in diminished cognitive flexibility caused by shifts from hippocampus-dependent memory to striatum-dependent memory [e.g., 58–62] and behavioral maladaptation due to amygdalar CRF-mediated excitation [e.g., 9, 29, 43, 55]. In addition, upon involvement of the nucleus accumbens (ventral striatum) that is associated with reward-related behavior, reinstatement of drug addiction via glutamate synaptic plasticity may occur [63]. This effect can be explained by the fact that in the nucleus accumbens, β-adrenoreceptors promote memory consolidation of positive and negative arousing experiences [64, 65]. It is thus not surprising that CRF-1 receptor antagonists are being considered as a promising pharmacotherapy for depression, anxiety, cognitive/neurodegenerative, and stress disorders [e.g., 66–68; see also 69–71].

## Effects of stress on neuronal mechanisms shared with cognitive and emotional processing

### Transcriptional and epigenetic effects, and interindividual genetic variation

*Summary of the concepts*: Psychological stress can induce various epigenetic effects that are associated with the activation of GR. The effects implicated in the pathogenesis of neurocognitive and psychiatric disorders. At the same time, polymorphism in genes associated with serotoninergic signalling has been linked to depression susceptibility, cortisol response to stressors, and greater risk for developing mild cognitive impairment.

*Takeaway*: Examining the impact of gene × environment interactions is superior to investigating interindividual genetic variation by itself.

#### Epigenetic regulation: central players

**Fig. 1:**
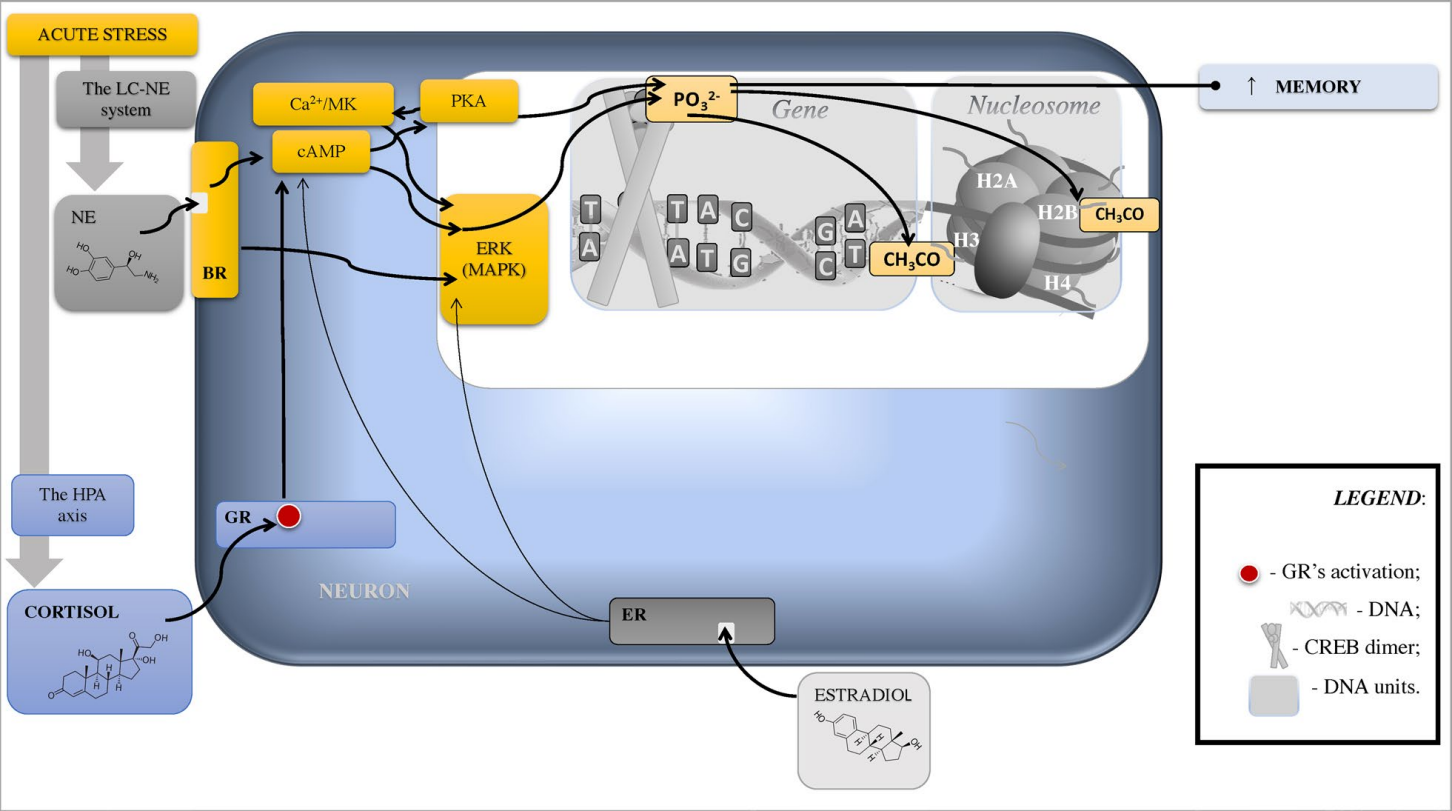
Epigenetic Mechanisms of Memory Alteration Following Acute Psychological Stress. Simplified and schematic model of indirect epigenetic mechanisms for fast (< 1 h) nongenomic effects induced by membrane-associate receptors during acute stress. Increased levels of NE bind to BR (namely β2) and rapidly increase Ca^2+^ influx and cAMP and Ca^2+^/MK activation, that triggers PKA to modulate synaptic activity (see Fig. 4) and phosphorylate CREB, which activates transcription and gene expression linked to neuronal plasticity, spatial memory, and long-term memory formation. This mechanism supports fear conditioning/learning, whereas severe stress can decrease initially activated Ca^2+^/MK, which relates to poor memory. Estradiol can activate ERs (mostly type β) linked to fear learning/conditioning via ERK/MAPK pathways which affect post-translational gene regulation via histone acetylation. In the amygdala, independently of sex-hormone levels, short photoperiod reduces melatonin and thus increases estradiol-induced phosphorylation of CREB (males) and ERK aka MAPK (females) linked to aggression. In acute stress, high estradiol levels in proestrus are linked to the prefrontal cortex memory deficit and altered glutamate signalling, potentially due to hyperactivated Ca^2+^/MK. Ca^2+^/MK, Ca^2+^/Calmodulin dependent kinases IIα; CH_3_CO, acetylation; CREB, cAMP response element-binding protein; ERK, extracellular regulated kinase, aka mitogen-activated protein kinase (MAPK); ER, estrogen receptors; GR, glucocorticoid receptor; H, histone; NE, norepinephrine; PO_3_^2^, phosphorylation; PKA, protein kinase A

**Fig. 2:**
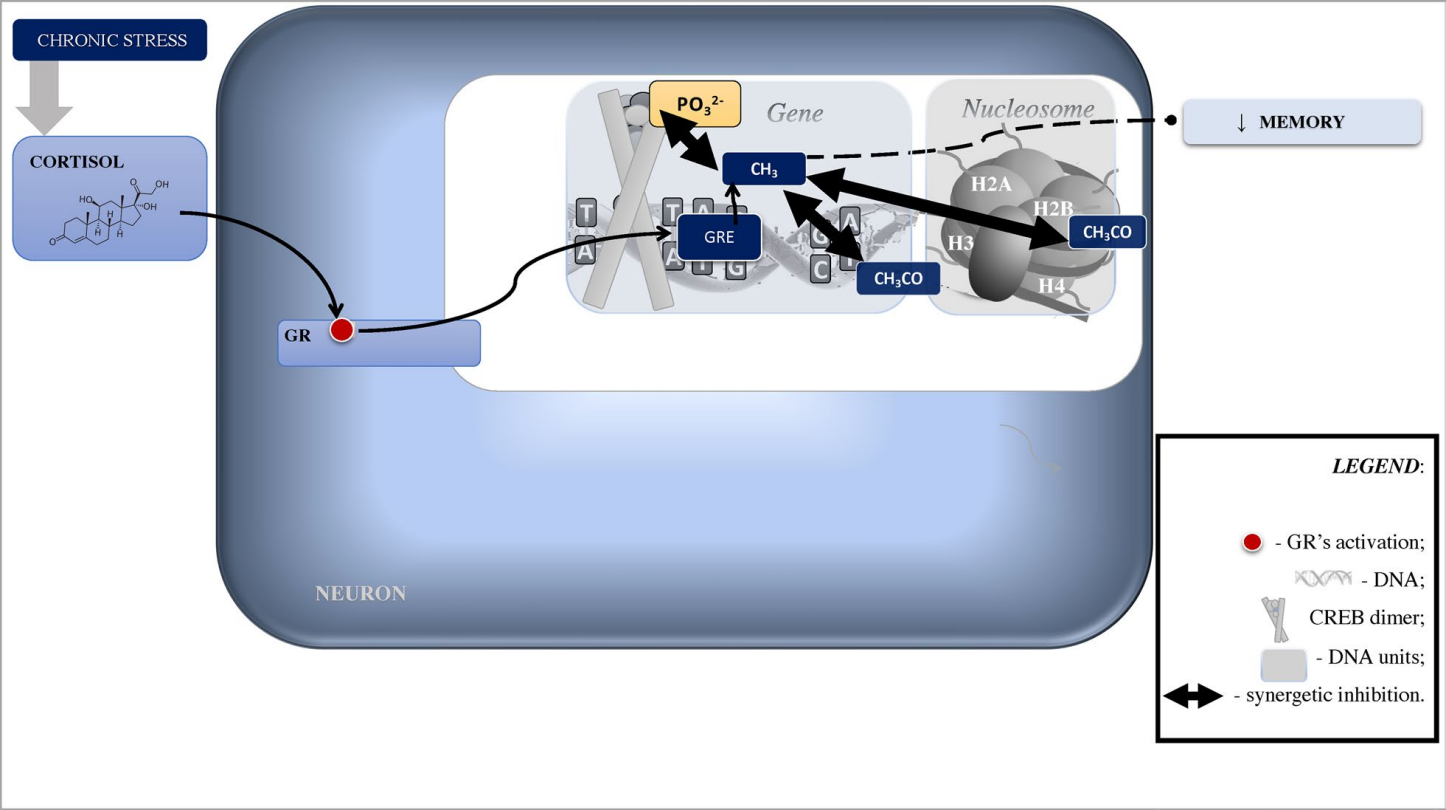
Epigenetic Mechanisms of Memory Alteration Following Chronic Psychological Stress. Simplified and schematic model of epigenetic mechanisms for slow (> 1 h) genomic effects induced by nuclear receptors. During chronic stress, increased nuclear levels of GRs-cortisol complex promote DNA methylation. Methylated DNA sites prevent CREB binding, and vice versa (depicted in double arrows as “synergetic inhibition”). At the same time, CREB activity largely correlates to histone acetylation which is essential for memory consolidation. CH_3_, methyl group; CH_3_CO, acetylation; CREB, cAMP response element-binding protein; GR, glucocorticoid receptor; GRE, glucocorticoid response elements; H, histone; PO_3_^2^, phosphorylation

Stress-induced surge in corticosteroid signalling can further initiate epigenetic changes (Figs. 1, 2), which have an influence on cognitive functions, behavior, and mood [e.g., 23, 72, 73; see also 74, 75]. The influence is facilitated by the GRs and mineralocorticoid receptors (MRs) cerebral distribution. GRs are highly expressed in the hippocampus and prefrontal cortex (PFC), which relates to executive functioning [e.g., 21, 28, 51]. Glucocorticoid secretion exhibits circadian and ultradian patterns with levels peaking in the morning and are essential for negative feedback after acute psychological stress [e.g., 76–81]. Whereas GRs assist information encoding (i.e., memorization), eradication of inadequate behavioral responses, and stress recovery, MRs, which are mainly engaged in the evening, facilitate processing of sensory information, analysis of environmental information, and execution of proper behavioral responses [28, 35, 48, 72, 82, 83].

#### Epigenetic regulation: factors underlying stress resilience

**Fig. 3:**
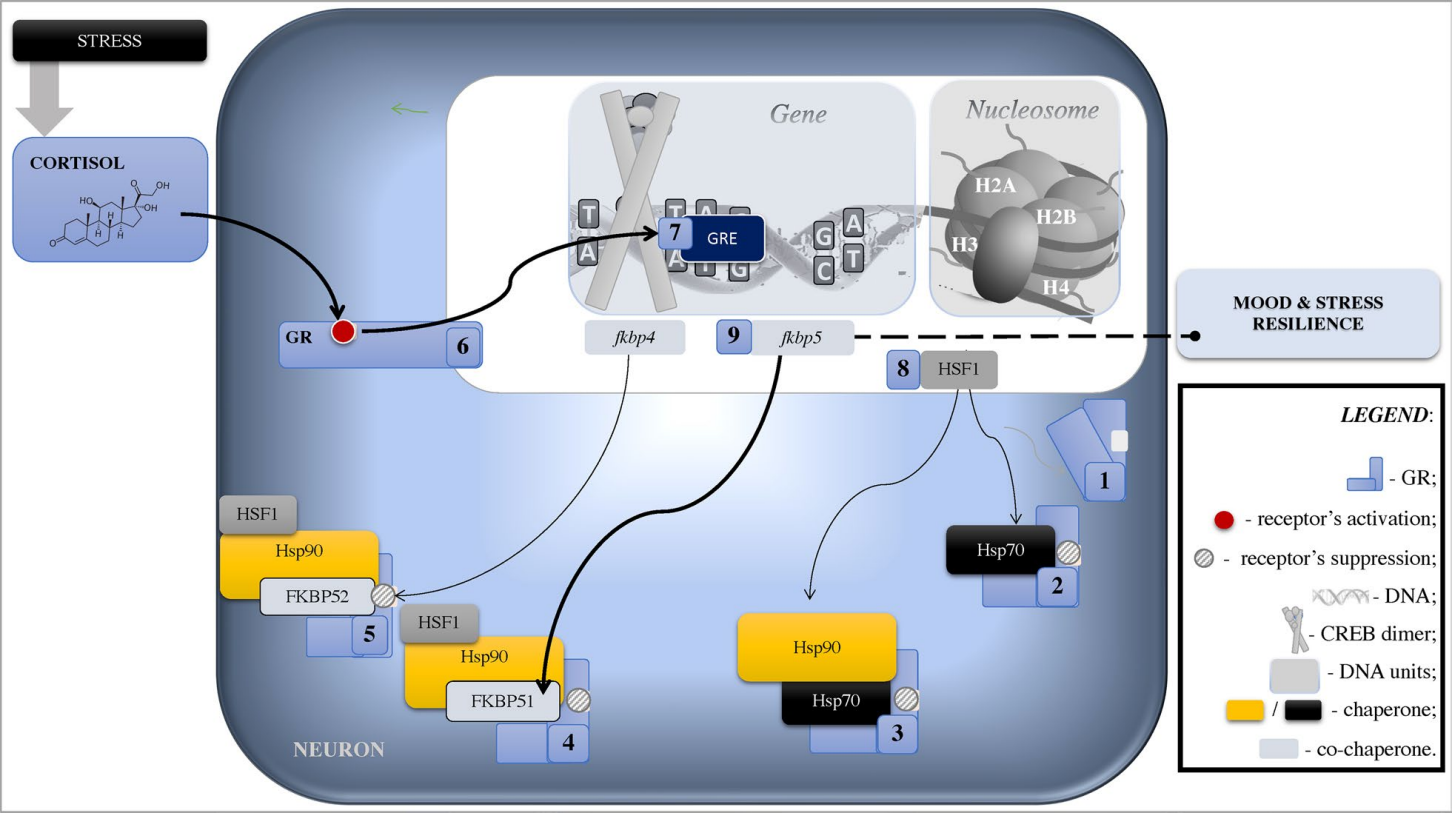
Epigenetic Mechanisms Underlying Stress Resilience to Neuropsychiatric Disorders. Stress increases levels of circulating cortisol that easily passes the cellular membrane. In the cellular plasma, [1] GR forms a complex with chaperones [2] Hsp70 (that partially unfolds and inactivates GR) and [3] Hsp90 (that facilitates GR maturation) [84, 87]. [4] Binding with co-chaperone FKBP51 (encoded by gene *fkbp5*) inhibits GR capacity for nuclear transactivation/signalling and detaches Hsp70 [35, 48, 99, 100]. [5] Encoded by gene *fkbp4,* co-chaperone FKBP52 competes with FKBP51 and its replacement increases affinity of the GR-Hsp90 complex to bind cortisol [84, 97]. [6] When cortisol binds, chaperone complex releases and thus GR and HSF1 translocate to the nucleus to initiate transcription linked to [7] memory formation [84, 87, 95, 101] and [8] gene expression for chaperones and co-chaperones that promote GR activation in stress-response [86, 94]. [9] In an ultra-short feedback loop, it also promotes gene *fkbp5,* which has close proximity to GRE. Polymorphism of *fkbp5* is associated with interindividual differences in stress reactivity. In addition, *fkbp5* expression increases with age due to decreased DNA methylation. This simplified model does not depict all protein/regulators involved in chaperone machinery and signalling pathways. CREB, cAMP response element-binding protein; GR, glucocorticoid receptor; GRE, glucocorticoid response elements; H, histone; HSF, heat shock factor 1

GRs depend on chaperones, especially heat shock proteins (Hsp) 70 and 90 [e.g., 84, 85]. Chaperones are multimeric complexes that control protein qualities (e.g., folding/unfolding, see Fig. 3) and protect cells by identifying emerging polypeptides from irreversible aggregation in the context of stress [e.g., 86, 87]. The chaperones dysregulated activity can result in misfolded and aggregated proteins like tau deposition, a hallmark of several neurodegenerative diseases [e.g., 86, 88–90]. Stress can affect the mechanisms of protein aggregation/clearance, which relates to proteotoxicity that is critical in the development and/or progression of neurodegeneration, including Alzheimer’s disease [e.g., 91–93].

#### Transcriptional and regulatory effects

**Fig. 4:**
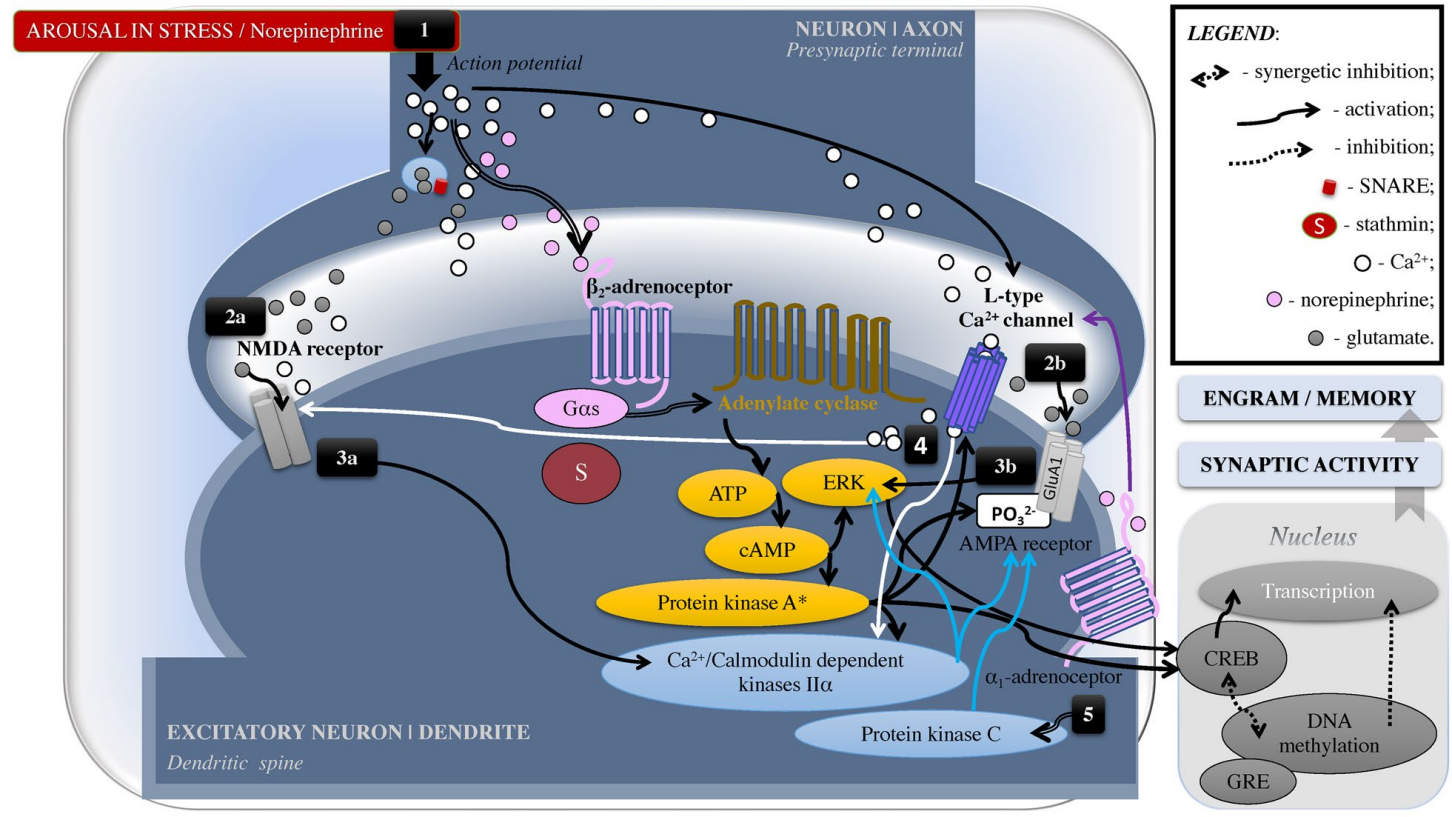
Psychological Stress: Noradrenergic Signalling and Synaptic Plasticity. A crucial part in memory formation belongs to synaptic activity/plasticity, particularly in the hippocampus. First, in the axon of a neuron, [1] action potentiation converts electrical stimuli into a chemical message [glutamate] to pass it through the synapse to the dendrite of another neuron. At the dendritic spine, ionotropic channels [2a] NMDA- and [2b] AMPA-type glutamate receptors “receive the message”. [3a] NMDA receptors facilitate Ca^2+^ entry that triggers Ca^2+^/Calmodulin dependent kinases Iiα, which results in synaptic incorporation of AMPA receptors—a necessary mechanism for long-term potentiation as a part of memory formation. [3b] Activated AMPA receptors stimulate ERK (aka MAPK). Additionally, in the dorsal hippocampus, activated estrogen receptors can stimulate GluA1 subunit via ERK linked to enhanced neurocognition in females but not in males. During acute stress (depicted by black double arrows), due to emotional arousal (e.g., fear), released norepinephrine binds to β2-adrenoreceptors (G-protein-coupled receptor) and activates protein Gαs; that stimulates adenylate cyclase and cAMP synthesis, which accumulation triggers protein kinase A and ERK/MAPK (depicted by yellow ovals, see Fig. 1 for more details). Protein kinase A activates AMPA receptor by its subunit GluA1 phosphorylation that results in the AMPA receptor’s trafficking and synaptic incorporation. As well, protein kinase A activates Ca^2+^/Calmodulin dependent kinases IIα directly and via [4] stimulation of L-type Ca^2+^ channel that increases Ca^2+^ influx and activates the kinases further (white arrows depict Ca^2+^ signalling). During severe stress, excessive norepinephrine release can also [5] activate α_1_-adrenoreceptors and trigger protein kinase C signalling that activates AMPA receptors. Stathmin is a microtubule-stabilizing and ERK-regulated protein that displays cytoprotective function. Specifically, dynamical changes in the microtubule stability are vital for synaptic plasticity and long-term potentiation. Synaptic input, such as during learning/facing a threat, hyperactivates stathmin, which decreases microtubule stability (depolymerization within first hour). SNARE protein complexes interact with serotonin signalling regulated by *fkbp5* gene (see Fig. 3), that potentially can determine an individual’s susceptibility to stress and depression. cAMP, cyclic AMP; CREB, cAMP response element-binding protein; ERK, extracellular regulated kinase, aka mitogen-activated protein kinase (MAPK); GR, glucocorticoid receptors; GRE, glucocorticoid response elements; PO_3_^2^, phosphorylation

**Fig. 5:**
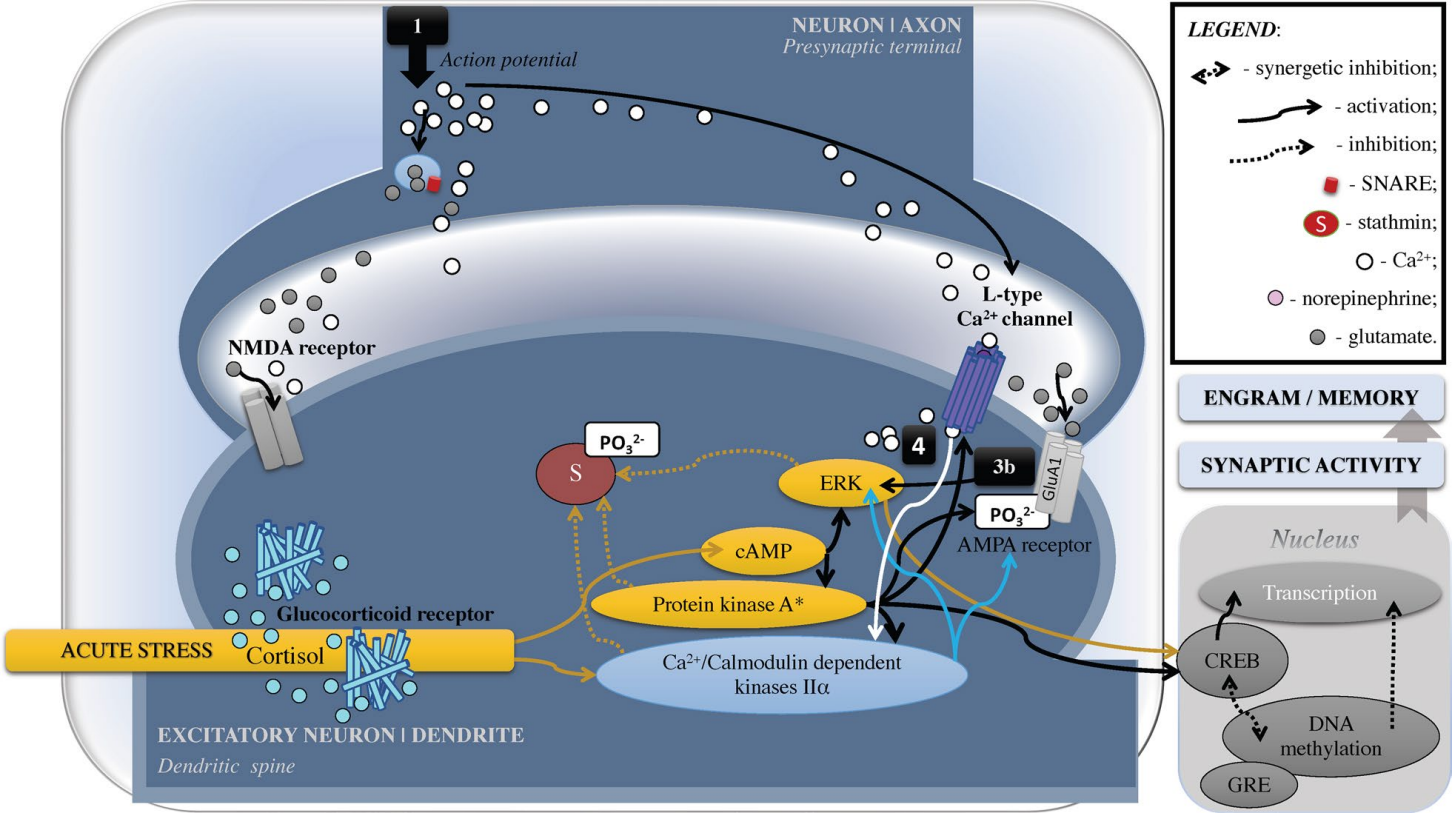
Acute Psychological Stress: The Role of Cortisol and Kinases in Memory Formation. During acute stress (depicted by mustard/dark yellow arrows), rapidly increased levels of cortisol can activate GRs that increase surface expression of NMDA and AMPA receptors (nongenomic memory effect). Upregulated Ca^2+^/Calmodulin dependent kinases IIα pathway activates CREB mechanism (fast indirect epigenetic effect related to neuropsychiatric outcomes such as anxiety and increased risk for post-traumatic stress disorder). At the same time, activated ERK/MAPK inhibits stathmin via phosphorylation; that stabilizes microtubules and, in turn, activates incorporation of the GluA2 subunit (AMPA receptor) to synaptic sites, which is necessary for long-term memory formation yet supports fear conditioning/learning. See Fig. 4 for the path [1]–[4]. cAMP, cyclic AMP; CREB, cAMP response element-binding protein; ERK, extracellular regulated kinase, aka mitogen-activated protein kinase (MAPK); GR, glucocorticoid receptors; GRE, glucocorticoid response elements; PO_3_^2^, phosphorylation

**Fig. 6:**
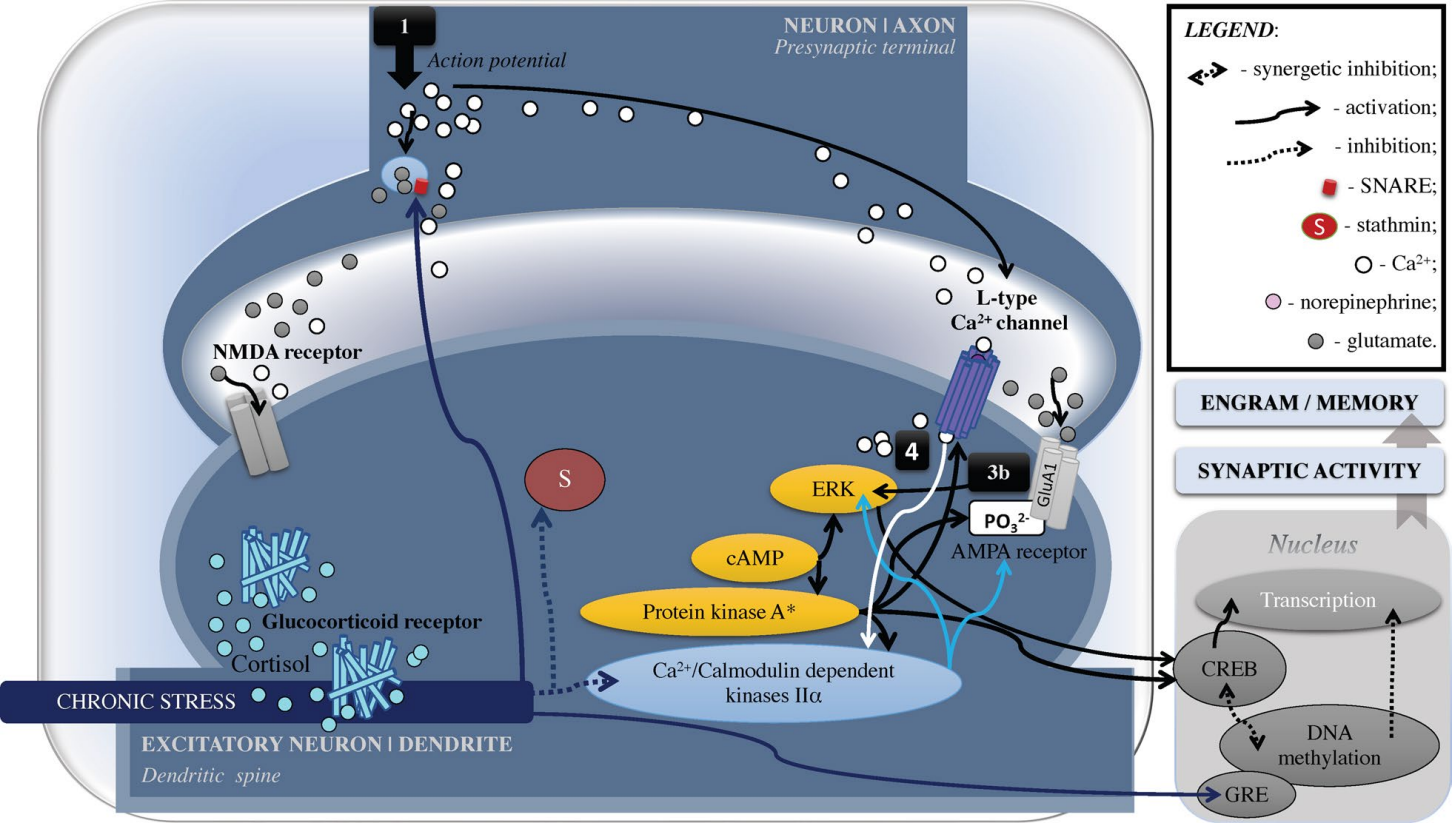
Chronic Psychological Stress: Proteinopathy and Synaptic Plasticity. During chronic stress (depicted by dark blue arrows), prolonged activation of GRs inhibits Ca^2+^/Calmodulin dependent kinases IIα and can lead to DNA hypermethylation that supresses gene transcription and protein synthesis (epigenetic memory effect). Severe stress can also reduce the expression of the stathmin that is related to the cellular skeleton, mitosis, and synaptic plasticity, which in turn, relates to poor learning and apoptosis. As well, chronic stress/single prolonged stress can increase SNARE complex formation but alter neurotransmitters fusion that relates to excitotoxicity and pathological accumulation of aggregated proteins. Alteration of the protein-kinase dynamics increases risk for proteinopathy and, in turn, depression and neurodegenerative disorders. See Fig. 4 for the path [1]–[4]. cAMP, cyclic AMP; CREB, cAMP response element-binding protein; ERK, extracellular regulated kinase, aka mitogen-activated protein kinase (MAPK); GR, glucocorticoid receptors; GRE, glucocorticoid response elements; PO_3_^2^, phosphorylation

The role of GRs in memory relates to their translocation ability in neurons. Specifically, GRs are dynamic molecular structures located mainly in the neural cytoplasm. GRs have the capacity to bind all steroid hormones with a ligand-binding domain that has 12 helices in the form of a “sandwich” [e.g., 94, 95]. Upon cortisol binding, activated GRs release Hsp90 that eases GRs’ nuclear translocation, where it acts as a DNA-binding transcription factor; GRs’ binding to DNA glucocorticoid response elements activates transcription—that is, transactivation effect seen in the increased rate of gene expression [e.g., 35, 48, 72, 84, 86, 87, 94, 96–100; see Fig. 3]. GRs’ nuclear translocation can also induce a transrepression, which is an epigenetic repression of other transcription factors including NF-κB and AP-1 related to the proinflammatory immune response [95, 101]. In addition, nuclear GRs can cause indirect genomic effect via cAMP-dependant protein kinase A that correlates to learning and memory function [e.g., 102; see Figs. 4–6].

Further, stress-activated GRs can induceDNA methylation via mitogen-activated protein kinase (MAPK), also known as extracellular signal-regulated kinases (ERK), that are required for fear memory and mediate stress-related behavioral effects of GRs [e.g., 103, 104; see also 105–108; Figs. 1–6]. The DNA methylation, which is a chromatin re-modelling by the addition of methyl groups (CH_3_) to cytosine nucleotide during transactivation (see Figs. 1, 2), serves as a “molecular bridge” between the external (i.e., environment) and internal (i.e., cellular) world [e.g., 109–114]. It is regulated by de novo methyltransferases that are crucial for hippocampal memory in contextual fear conditioning, late-phase long-term potentiation, and spatial memory [e.g., 115].

Another important mechanism that can be engaged in stress-associated neuropsychiatric outcomes belongs to the nucleosome modifications. A nucleosome is a “bead” segment of DNA wrapped around histones, which are octamers of duplicated H2A/H2B dimer and H3/H4 tetramer proteins. Histones display a globular structure with protruding “tails” from the nucleosome—N-terminal domains, exclusively in H2A and H2B extra C-terminal domains (Figs. 1, 2). The nucleosome has dynamic phases of “breathing” when it unwraps and rebinds DNA, and thus transiently exposes DNA sites [e.g., 116] so that genes can be regulated post-translationally via (but not limited to) methylation. The “breathing” has a dual role: it condensates DNA to deactivate transcription, and relaxes DNA to activate transcription [e.g., 117, 118]. This process modifies histone tails with “postal codes” (i.e., molecular tags) to be delivered and recognized by “reader” proteins involved in chromatin re-modeling [e.g., 115, 118]. The molecular tagging of the histone tails is an epigenetic mechanism of learning and memory, which is regulated by “eraser” proteins that remove established histone-tail modifications [119]. However, stress can block this process via histone acetylation mechanisms and, in turn, lead to depressive- and anxiety-like behavior, visceral hypersensitivity, and cognitive deterioration [e.g., 120–123]. The reason is that histone acetylation is a rapid process of histone modification. It is regulated by histone acetyltransferases, which are both the histone “postal codes writers” and “postal codes readers” balanced by histone deacetylase activity that may reverse histone acetylation [e.g., 119]. Histone acetyltransferases contribute to the transcriptional control during memory formation, including contextual fear conditioning, novel object recognition, spatial memory, and long-term potentiation [e.g., 115, 124, 125]. Additionally, histone acetylation and epigenetic mechanisms of de novo DNA methylation are synergetic. For instance, histone deacetylase inhibitor infusion via transcription factor (*NGFI-A*) binding to the GR promoter reverses DNA methylation, GR expression, and HPA reactions [e.g., 114, 126]. Furthermore, inhibition of DNA methyltransferase blocks promoted histone H3 acetylation in contextual fear conditioning [e.g., 115, 127]. It is thus not surprising that histone modifications are implicated in the pathogenesis of cognitive dysfunction, neuropsychiatric conditions, and neurodegenerative disorders such as PTSD and Alzheimer’s disease [e.g., 128–130]. Accordingly, inhibitors of histone deacetylase show neuroprotective properties and show potential as an intervention in the treatment of neurocognitive disorders and PTSD [e.g., 119, 131, 132].

Stress can also alter methylation of hippocampal brain-derived neurotrophic factor (BDNF), which plays an important role in adaptation to chronic stress by promoting neuroplasticity [e.g., 133, 134]. Prolonged cortisol hypersecretion in chronic stress increases methylation of the hippocampal BDNF promoter, which is positively correlated to memory consolidation in contextual fear learning [e.g., 111, 135; also see 136–139]. Chronic stress also supresses BDNF expression that results in subsequent hippocampal atrophy and deficient acquisition/consolidation of verbal declarative memory [e.g., 140–143]. Reductions in hippocampal BDNF are linked to desensitization of GRs, higher stress vulnerability, and predisposition to psychiatric comorbidity in stress [e.g., 133, 144–146]. The mechanism involves a downregulation of peroxisome proliferator-activated receptor δ, which is related to significantly reduced neurogenesis and behaviors that are consistent with depression [147].

At the same time, stress-related changes in the epigenome can be reversed. For instance, glutamate receptor (NMDA) blockade inhibits BDNF expression and prevents its methylation, which in turn supresses altered memory formation [e.g., 111]. Moreover, the induction of gene-specific demethylation (e.g., by genetic removal of a regulator of active DNA demethylation or with DNA methyltransferase inhibitors) can improve late-phase hippocampal potentiation, spatial memory, and contextual fear memory consolidation [e.g., 115; see also 10]. At least one systematic review and meta-analysis revealed that patients with PTSD have elevated serum BDNF levels compared to healthy individuals [148]. Despite equivocation in the literature, some clinical studies have related the BDNF Val66Met polymorphism to modulation of stress sensitivity [e.g., 149]. For instance, the BDNF Val66Met polymorphism has been shown to moderate the relation between PTSD and fear extinction learning [150] and aversive memory bias in women with PTSD but not in psychiatrically healthy women [151]. At the same time, PTSD and dementia have been shown to be bidirectionally linked [e.g., 152], and that PTSD is a potentially modifiable risk factor for dementia [153; see also 69].

#### Interindividual genetic variations

Although stress can initiate epigenetic changes that modulate susceptibility to neuropsychiatric conditions, a glucocorticoid-dependent encoding gene *fkbp5* plays a major role in stress resilience [e.g., 84, 96, 154]. The *fkbp5* gene is in close proximity to a glucocorticoid-responsive element [96, 155, 156]. Moreover, the *fkbp5* polymorphism correlates with activation of GRs and can thus increase post-traumatic stress susceptibility to psychiatric disorders [99, 101, 157]. The *fkbp5* gene, which encodes co-chaperone FKBP51 and regulates serotoninergic signalling [e.g., 158], is associated with depression susceptibility and reduced cortisol response to stress due to GRs hypofunction. For example, *fkbp5* rs1360780 T‐allele carrier status is related to GR resistance seen in reduced concentrations of plasma cortisol and ACTH following dexamethasone administration in depressed patients compared to healthy controls [159]. In healthy volunteers, the *fkbp5* polymorphism modulates recovery to the Trier Social Stress Test (TSST). Specifically, the *fkbp5* variants (rs1360780, rs3800737, and rs4713916) were related to less cortisol recovery and higher anxiety after psychosocial stress, whereas the GR polymorphism (Bcl1 but not N363S) was related to anticipatory cortisol levels [160]. Additionally, there is a male-specific effect in *fkbp5* polymorphism (rs3800737) that is linked to the cortisol responses to TSST (i.e., peak response and response area under the curve), whereas variants rs7209436 and rs110402 in CRH receptor gene (*CRHR1*) are associated with a trait anxiety × baseline cortisol interaction [161].

The polymorphism in *fkbp5* has also been shown to predict individual differences in stress-related memory deficits [e.g., 162, 163] and functional activity/connectivity of the amygdala related to emotional (anxiety/depression) responses to threat [e.g., 164–168]. Its interaction with early life stress is also predictive of reduced connectivity between the amygdala and parahippocampal gyrus, caudate, and frontal gyri [169; see also 170]. In healthy youth, for example, *fkbp5* genotypes (rs7748266, rs1360780, rs9296158, rs3800373, rs9470080 and rs9394309) predicted relatively increased threat-related dorsal amygdala reactivity in the context of higher self-reported emotional neglect [171]. Genetic variants in *fkbp5*, as well as CRHR1 and NR3C2, have been also found to be associated with higher HPA activity, and their interaction with early life stress is associated with right amygdala reactivity to threat and anxious arousal [172]. In adults exposed to high levels of childhood trauma, rs3777747, rs4713902, and rs9470080 (main effects) and rs3800373, rs9296158, and rs1360780 (interactive effects with Childhood Trauma Questionnaire score) were related to a greater risk of a lifetime suicidal behavior [173].

There are interindividual and sex-specific peculiarities of the stress-response related to FKBP51 functions. Specifically, FKBP51 interacts with sex hormones (progesterone and androgen) receptors that modifies their sensitivity. As well, FKBP51 competes with FKBP52 regulation of memory-associated synaptic plasticity and transmembrane calcium channels [e.g., 84, 96]. In healthy undergraduate students, exposure to a socially evaluative cold pressor test directly prior to verbal learning reduced immediate verbal recall in *fkbp5* risk allele carriers (rs1360780, rs3800373 and rs929615). In contrast, the stress task enhanced 24-h later recall and recognition memory in non-carriers of the risk alleles [163]. In young healthy adults, *fkbp5* A-allele carriers had less pronounced autonomic responses to stress and poor working memory performance on the Stroop color-word task (the results of which positively correlated to the degree of self-reported early life adversity), which was associated with poor health behaviors when compared to *fkbp5* GG homozygotes [162].

More studies are needed, however, to investigate serotonin (5-HT) signalling and interactive effects of genetic polymorphisms in relation to the processing of specific memory types. Indeed, spatial memory versus emotional memory, or memory formation versus memory retrieval depend on different circuits and signalling pathways, and therefore may be differentially affected across distinct types, stages, and phases of stress. Such inquiry is also needed given that 5-HT receptors such as 5-HT1A, 5-HT2A, and 5-HT7 play an important role in learning and memory [e.g., 174–182]. In addition, there is an association between the serotonin transporter-linked promoter region (*5HTTLPR*) polymorphism and clinical manifestations in neurodegeneration—for example, cognitive impairment variations in Alzheimer’s disease and delusions in Lewy body dementias [e.g., 183–186]. The evidence suggests that the *5HTTLPR* variants modulate the levels of neural activation in the hippocampus and PFC during memory retrieval in acute psychosocial stress [184; see also 187] and can have a negative effect on memory via mediation of the HPA axis in the elderly [e.g., 188]. These data can explain the findings that the *5HTTLPR* polymorphism is a risk factor for developing mild cognitive impairment, which precedes Alzheimer’s disease [e.g., 189]. Therefore, it is one of the neurogenetic components of stress resilience and neuropsychiatric outcomes [e.g., 168, 190, 191].

### Neurotransmitter signaling

*Summary of the concepts*: Stress-induced GR levels modulate neuronal plasticity and stress resilience via neurotransmitters:

- Successful coping under stressful conditions relates to glutamatergic signalling.
- During stress, emotional arousal can alter Ca^2+^/Calmodulin-dependent kinases involved in emotional and cognitive processing.
- The protein-kinase dynamics can play an important role in emotional homeostasis and cognitive reserve that may determine an individual’s susceptibility to neuropsychiatric disease.
- Stress-induced increase in norepinephrine (NE) levels affect memory in inverted U-shaped relation.
- Polymorphisms in genes associated with dopamine levels can be a prospective screening tool for stress resilience capacity based on the phenotypic variability of behavior and cognition patterns.

*Takeaway*: Pharmacotherapy based on neurotransmitter signaling and administrated to block harmful effects of glucocorticoids on working memory could eliminate memory consolidation of emotionally significant experiences.

#### Glutamate

During stress, hippocampal-dependent memory and learning can be influenced by emotional hyperarousal (i.e., anger, fear, or happiness). This phenomenon involves amygdala-mediated effects on memory consolidation and retention [192; see also 10, 193–197], which is a reinforcement of synaptic networking between neuronal ensembles (engrams) [e.g., 198, 199]. Engrams require dynamic variability (i.e., synaptic plasticity) that occurs during synaptic transmission: as hyperarousal initiates presynaptic input (i.e., a neurotransmitter release) and postsynaptic output (i.e., receptors expression), it boosts engrams [200]. This is how the amygdalar basolateral nucleus redirects attention toward aversive stressors (i.e., attentional bias), promotes encoding and consolidation for aversive memory, consolidates contextual and spatial information, and fortifies memory of novel contexts [e.g., 36, 192, 201–211]. Hyperarousal in stress thus enables time-dependent associative memory, especially for aversive information, which inhibits or eliminates previous memory and facilitates defensive behavior. Both maintenance and inhibition of associative memory depend on synaptic plasticity and magnitude of hyperarousal [e.g., 212]. Although initial emotional experience mediates plasticity, prolongated stress can suppress hippocampal plasticity and thus impair hippocampal processing [e.g., 192, 201, 213–217].

The activation pattern of persistent changes (increase/decrease) in synaptic transmission defines the communication between synapses, which can be in the form of long-term potentiation (upon increase) or long-term depression (upon decrease). Long-term potentiation follows presynaptic glutamate release and subsequent postsynaptic depolarization. The activation pattern in synaptic transmission is mediated by ionotropic (cation) channels, the NMDA- and AMPA-type glutamate receptors, often via AMPA-receptor subunit composition [e.g., 218–220].

Glutamate is the major excitatory neurotransmitter of the central nervous system, with exceptionally high neuronal levels. Stress-activated GRs can increase glutamate release via pre- and post-synaptic mechanisms, which is a risk for hyperactivation of the glutamate receptors [i.e., excitotoxicity; 4, 221–223]. The AMPA receptors are transmembrane channels composed of subunits GluA1–4 that differ by intracellular C-terminal tails [e.g., 224]. Each AMPA receptor subunit has a site that can bind glutamate; when two sites are occupied with glutamate, the receptor’s pore (cation channel) opens and enlarges with an increase of occupied binding sites [e.g., 225]. Released from the Golgi apparatus into the synaptic membrane, subunits of the AMPA receptors are reserved to initiate long-term potentiation due to their capacity for rapid redistribution from non-synaptic sites to the synapse, which is necessary for synaptic plasticity: if the postsynaptic AMPA receptors are inactive, synapses are silent [e.g., 224]. Distribution of the AMPA receptors on the synaptic surface is bidirectionally regulated by protein interactions. Of particular interest are scaffolding proteins PSD-95 and SAP97 that regulate incorporation (i.e., postsynaptic density) and function of the AMPA receptors [e.g., 226], which is an essential process for both synaptic transmission and plasticity that underlines learning and memory [e.g., 227–229].

Chronic stress can reduce expression of the AMPA and NMDA receptors at the synaptic membrane due to their post-translational modification, such as increased ubiquitin–proteasome-dependent degradation of the receptor’s subunits [e.g., 223; see also 10]. However, stress can also increase the hippocampal expression of GluA2 subunits linked to enhanced spatial learning and memory [e.g., 230, 231]. Researchers have also shown that higher fear-memory retrieval in adult rats is associated with the amygdala-driven preferential upregulation of PSD-95 and GluA2, contrary to reduced fear-memory retrieval in juvenile rats that is linked to the upregulation of kinases (PKMζ and PI3K), GluA2/3 and GluA1 in the dorsal hippocampus [232]. Stress-activated GRs can also alter glutamatergic signalling via increased GluA1 phosphorylation (at Ser 831) that synchronizes with increased phosphorylation of the main NMDA receptor subunits (NR-1 and NR-2B) as integral functioning of the AMPA and NMDA receptors [233]; see also 234]. Successful coping under stressful conditions is determined by integral AMPA/NMDA receptor function [233–238], whereas altered glutamatergic signalling is strongly associated with psychiatric disorders and Alzheimer’s disease [e.g., 239, 240].

#### Calcium

Physiological and psychological arousal is modulated by the integration of interoceptive and exteroceptive inputs and is an essential factor for attention and cognitive processing. Calcium (Ca^2+^) influx is necessary for behavioural arousal states, and is mediated by NMDA receptors which facilitate Ca^2+^ entry which triggers Ca^2+^/Calmodulin-dependent kinases (Ca^2+^/MK) to shape synaptic structure by the AMPA-receptors’ redistribution—that is, translocation into the synaptic membrane (see Figs. 4, 5, 6). This receptor’s redistribution promotes actin cytoskeleton involved in the dendrite (its spine) and axon development that is necessary for hippocampus-dependent memory [e.g., 241, 242]. Thus, hippocampus-dependent learning and memory require Ca^2+^ influx, which is supported by several types of channels: (1) neural, high-voltage-activating aka N-type or Non-L involved in certain forms of LTP [e.g., 243]; (2) residual, immediate-voltage-activating aka R-type such as Ca_v_2.3 α_1E_ subunit involved in spatial memory formation [e.g., 244]; and (3) transient, low-voltage-activating aka T-type such as Ca_v_3.2 subunit involved in context-associated memory retrieval [e.g., 245]. The Ca2^+^ channels that are most highly distributed in dendrites are long-lasting, high-voltage-activating—that is, “triggered by strong and sustained depolarizations,” aka L-type Ca2^+^ channels, which regulate “gene expression, synaptic efficacy, and cell survival” [246; see also 247].

Emotional arousal at the time of memory consolidation that promotes memory maintenance relates to intracellular Ca^2+^ influx via L-type of Ca2^+^ channels, and Ca2^+^ concentration is regulated mainly by Ca^2+^/MK in the amygdala, hippocampus, and PFC. Electrochemically, it is seen at the arrival of an action potential that triggers Ca^2+^ entry at the presynaptic neuronal terminal, which further activates vesicles with neurotransmitters (e.g., NE, glutamate, GABA, dopamine, serotonin) to release into the synaptic cleft (see Fig. 4). Released neurotransmitters join the postsynaptic receptors, resulting in the synaptic transmission that can be excitatory (mainly glutamate-mediated and formed in dendritic protrusions, aka spines) or inhibitory (mainly GABA-mediated) membrane potential [e.g., 218; see also 248–250]. During memory retrieval, Ca^2+^/MK activate proteasome-mediated postsynaptic protein degradation—and thus labialize/weaken memory due to reduced synaptic efficacy—whereas during memory reconsolidation, Ca^2+^/MK reroute synthetized de novo proteins to more active synapses [e.g., 251].

In the context of stress, activated GRs can regulate mitochondrial function of Ca^2+^ holding capacity by translocating into mitochondria as a complex with the anti-apoptotic protein Bcl-2. This mechanism was found to be correlated to neuronal plasticity and stress resilience in an inverted “U”-shape depending on corticosterone levels. Whereas low doses improve neural viability/resilience, high doses (i.e., as it is observed in intense stress) augment kainic acid-induced toxicity of cortical neurons [252, see also 253], which partially explains the toxic neural effect of intense and chronic stress.

Chronic stress is also associated with dysfunction of the Ca^2+^/MK cascade, which is seen in increased intracellular Ca^2+^ concentration and reduced Ca^2+^/MK IIα expression in the medial PFC [e.g., rat models of PTSD, 254]. Intracellular Ca^2+^ elevation for a prolonged period is toxic and is associated with Ca^2+^-dependent cellular mechanisms that alter dendritic spine density and neuronal morphology of the pyramidal neurons in the medial PFC [255; see also 256].

In addition, Ca^2+^/MK IIα is linked to cAMP response element-binding protein (CREB) activation (Figs. 1–6), which in turn inversely correlates to the amygdala-induced anxiety-like behavior [257, also see 258–261] and altered CREB-BDNF signaling pathway relates to cognitive decline and Aβ toxicity in Alzheimer’s disease [260]. Antidepressants can regulate hippocampal Ca^2+^/MK IIα an adaptive way: namely, whereas short-term treatment inhibited the kinase activation in a concentration-dependent manner, chronic treatment up-regulated Ca^2+^/MK IIα [262]. Furthermore, in a mouse model of chronic social defeat stress, anxiolytic effects have been associated with the ERK/CREB/BDNF signaling pathway [263]. Inhibited ERK activity in the hippocampus is a promising therapeutical target for depression [e.g., 264, 265]. In support of this possibility, treatment with resveratrol to prevent stress-induced cognitive deficits relates to the upregulation of CREB/BDNF expression in the hippocampus in vivo and in vitro (i.e., in a rat model of chronic unpredictable mild stress; [266, see also 267, 268].

#### Protein-kinase dynamics

Stress resilience related to glutamatergic transmission involves several protein complexes, most notable of which is the soluble *N*-ethylmaleimide-sensitive factor attachment protein receptor (SNARE) complex that regulates vesicular neurotransmitter release (i.e., fusion) in a final step of presynaptic vesicle traffic [e.g., 269–271, see Figs. 4, 5, 6]. The activity of SNARE complex potentially relates to hippocampal “protein adaptation” and emotional homeostasis, and thus may determine an individual’s susceptibility to stress and depression [e.g., 271–273]. Further, chronic antidepressant treatment (i.e., with fluoxetine, reboxetine, or desipramine) has been found to increase neurogenesis and reduce stress-induced presynaptic glutamate release linked to altered memory. Moreover, the mechanism underlying this effect is potentially associated with assembly of SNARE complex [e.g., 221–223, 274–278].

Accumulating evidence suggests that the kinases (e.g., Ca^2+^/MK IIα) and proteins (e.g., SNARE protein complexes) play an important role in emotional homeostasis and cognitive reserve and may thus determine an individual’s susceptibility to neuropsychiatric disorders. We hypothesize that stress resilience thus relates to protein-kinase dynamics linked to cellular toxicity and apoptosis and, in turn, neurodegeneration. Specifically, in acute and intense stress, increased NE influx with emotional arousal appears to skew the interplay toward altered Ca^2+^/MK IIα expression/activation that can affect the CREB pathway (Fig. 5), which relates to fear conditioning, anxiety, and attentional tunneling, and is potentially a risk factor for PTSD. In the context of severe chronic stress, the protein-kinase dynamic is potentially skewed toward proteinopathy of SNARE protein complex (related to glutamate-induced excitotoxicity, reviewed above) and stathmin (related to the cellular skeleton and mitosis) that impair synapses (Fig. 6).

##### Acute stress and Ca^2+^/MK IIα

In a rat model of PTSD, single prolonged stress (SPS) exposure altered free intracellular Ca^2+^ levels (initially increased but then decreased), increased calmodulin expression, and decreased Ca^2+^/MK IIα expression in the medial PFC (day 1 after SPS) [254]. Similar findings were obtained for the basolateral amygdala [279] and hippocampus [280]. Calmodulin and Ca^2+^/MK IIα expressions have also been shown to initially increased but then decreased after SPS in the dorsal raphe nucleus, which was assumed to be associated with the activation of 5-HT1A receptor (related to neuronal inhibition via suppression of adenylyl cyclase, Ca^2+^/MK IIα, and AMPA receptors) [281; see also 282–284].

##### Severe chronic stress and proteinopathy

Recent findings on hippocampal presynaptic membrane dysfunction in rat models of PTSD demonstrated that SPS disabled synaptic vesicle fusion (i.e., reduced expression of synaptotagmin-1, the calcium-ion sensor for fusion), extended axon (i.e., increased expression in proteins, e.g., tau and β-tubulin, but decreased expression in p-tau and stathmin), and potentially increased SNARE complex formation (i.e., increased VAMP, STX1A, and Munc18-1 expression) [273]. The formation of SNARE protein complexes interacts with serotonin signalling regulated by *fkbp5* gene associated with depression and low cortisol response to stress. In addition, SNARE protein complexes apparently play an important role in pathological accumulation of aggregated proteins, for instance, α-synuclein in Parkinson’s disease [e.g., 285].

Another important protein is stathmin (aka oncoprotein 18) that regulates stability of the cellular cytoskeleton (i.e., destabilizes microtubules which have assembling-disassembling dynamics) and cycle (e.g., cell proliferation and accumulation) [286, 287, see also 288]. Stathmin also displays a cytoprotective function (e.g., during cellular/osmotic stress) [289], which is associated with cAMP-dependent protein kinase signaling [e.g., 290]. These are significant synaptic factors, and stathmin mutation/reductions relate to impaired memory, fear recognizing/processing/learning, and altered behavior [e.g., 291–296]. Altered expression in stathmin genes is associated with anxiety, poor learning, fear memories, and PTSD in animal models of stress [e.g., 297, 298; see also 299]. In one study, SPS reduced expression of stathmin in the hippocampus, medial PFC, and amygdala [294]. At the same time, stathmin alteration is implicated in neurodegeneration, such as Alzheimer’s disease [e.g., 297, 299, 300], while stress can escalate apoptosis [e.g., 301]. The pathways of apoptosis can include but are not limited to: (1) enzymes facilitating target proteins (e.g., receptors); (2) MAPK pathway; and (3) GRs [e.g., 302]. Additionally, recall that chaperones, which, when dysregulated by stress, can also lead to abnormal protein properties. As a result, accumulated protein aggregation into toxic non-native oligomers or fibrils/plaques (i.e., proteotoxicity) increases the risk for neurodegenerative disease. Conversely, behavioral stress-resilience (i.e., better coping) has been found to be associated with lower tau levels in older, amyloid positive adults without cognitive deficits [91].

Thus, increased levels of cortisol during acute stress can increase Ca^2+^/MK IIα expression /activation (Fig. 5), whereas chronic stress can decrease Ca^2+^/MK IIα expression/activation (Fig. 6). The stress response is regulated by 5-HT signaling via the ERK pathway associated with anxiety and PTSD due to 5-HT1 and 5-HT2A receptors’ competitive interaction [e.g., 178, 284]. This complex cellular dysfunction in enzymes and proteins can explain the molecular pathway intersection between depression and anxiety, as well as stress-induced neurodegeneration, especially when accounting for the fact that Ca^2+^/MK regulate apoptosis [e.g., 303], and similar to MAPK/ERK, are linked to tauopathies [e.g., 304–306].

#### Noradrenaline/norepinephrine

In acute stress, activation of the noradrenergic (LC-NE) system supports attention to the stressors [e.g., 1]. However, the noradrenergic signaling cascade can alter memory through activated cAMP-dependent protein kinase A via β-adrenoceptor activation, which is a fast response in contrast to a classical activation of GRs [e.g., 51, 206, 211, 307, 308]. Belonging to group A of the G protein-coupled receptor (GPCR) family, the β-adrenoceptor has a signature seven-transmembrane configuration [e.g., 309] in which cAMP/protein kinase A signalling pathways are shared with GR signalling [51, 205, 206, 310; see Figs. 1 and 4]. Activation of β-adrenoceptors with elevated levels of NE leads to phosphorylation of AMPA receptor’s subunit GluA1 at the Ser 818 site which promotes the AMPA receptor’s functions essential for long-term potentiation [e.g., 310]. Subsequent synaptic incorporation of Ca^2+^-permeable receptors (AMPA receptors containing GluA1, but missing GluA2) increases synaptic functionality [e.g., 220]. The distinctions are that amygdalar β-adrenoreceptors signalling activates AMPA receptors required for memory acquisition and working memory during threat, and then ERK/MAPK signalling facilitates fear memory consolidation through gene transcription and protein synthesis [e.g., 311; see also 312]. Remarkably, glucocorticoids influence deficit of working memory and enhancement of memory consolidation via a common activation of the noradrenergic signaling pathway within the medial PFC. An inverted U-shaped relationship between NE levels and working memory has been observed [e.g., 313].

Further, the LC neuronal activity regulates attention and low tonic activity relates to drowsiness and disengagement, whereas moderate-to-high tonic activity relates to arousal/hyperarousal [e.g., 314; see also 1]. In states of arousal without stress (i.e., an alert state without hypercortisolemia), released NE optimizes working memory via α_2A_-receptors. However, during a stress state, elevated NE levels impair PFC function and memory performance via α_1_- and β_1_-receptors [e.g., 315]. There is an inverted U-shaped dose–response relation that depends on the severity of the stressor and the condition that is observed in: (1) impaired memory for stressful life events with cortisol suppression, (2) timing-dependent learning improvement with moderate increase in cortisol concentration, and (3) impaired consolidation and recall in hypercortisolemia [e.g., 51, 211]. Therefore, pharmacotherapy (e.g., GRs and α_1_-/β-adrenoceptor antagonists, or protein kinase A inhibitors) administrated to block harmful effects of glucocorticoids on working memory can eliminate memory consolidation of emotionally significant experiences [e.g., 51, 315].

#### Dopamine

Stress-induced activation of the HPA axis increases dopamine neurotransmission in the PFC and activation of the downstream signalling cascades that negatively impact mood and cognition [e.g., 316; see also 317–320]. Indeed, it has been shown that emotional stimuli can influence working memory processing, manipulation, and maintenance that depend on the dorsolateral PFC, which is inversely coactive with the ventrolateral PFC [e.g., 321] and involve dopaminergic D_1_-receptors signalling. Like the NE effect, dopamine levels alter working memory in a U-shaped manner [e.g., 315, 322]. Furthermore, we consider polymorphism in genes associated with dopamine levels to be a prospective screening tool for stress resilience capacity based on the dopamine-related phenotypic variability of behavior and cognition patterns.

For example, the Val^158^Met polymorphism of catechol-O-methyltransferase (COMT, which is related to dopamine catabolism in the PFC and is regulated by estrogen) contributes to the complex Sex × Gene × Environment interactions affecting dopamine-dependent neurocognition and anxiety [e.g., 323, 324]. The Val^158^Met polymorphism has been found to influence decision-making following stressful life events [325]. As compared to the Val allele, the Met allele is linked to better working memory performance (including verbal, visuospatial, and novel social tasks) and poorer executive functioning, which is related to reduced dopamine levels in the PFC in children, adolescents, and adults [326, 327; see also 328]. COMT Val-allele load (COMT Val > Met), in turn, has been shown to be related to the dorsolateral PFC activity during working performance, such as encoding of new information, as well as temporal updating operations, but not in its subsequent retrieval. The distinctions are that high working memory load activates the PFC-parietal-striatal network, where activity in the right dorsolateral PFC is lesser in Val homozygotes than in ValMet individuals, but intermediate in Met homozygotes [329]. Relatively better performance in working memory is associated with decreased coactivity between the dorsolateral PFC and ACC for Val/Val genotype, but increased coactivity between the dorsolateral PFC-amygdala/hippocampus for Met/Met genotype. This association correlates to the regional cerebral blood flow in the amygdala and hippocampus for Val/Val, the parietal lobe for Val/Met, and the thalamus for Met/Met [330].

Resaerchers have also found that COMT is associated with emotional dysregulation and that dopamine receptor 2 genes can be a promising target of antipsychotic medications [e.g., 331]. Additionally, for monoamine oxidase A, H allele carriers have greater stress effects on the right anterior hippocampus hypoactivity and cortisol hyperresponse as compared to the L allele carriers [332]. At the same time, the monoamine oxidase A polymorphism predicts aggressive and oppositional behavior, with better working memory capacity related to fewer externalizing symptoms in children and adolescents [333]. Polymorphism in genes associated with dopamine levels can also serve as genetic biomarkers for higher risk of developing Alzheimer’s disease [e.g., 334–336].

## Sex hormone co-signaling

*Summary of the concepts*: Estrogen and androgen signalling influence memory and behavior, which is linked to stress resilience.

*Takeaway*: Estrogen effects are complex and play a dual, neuro-protective and neuro-harming role, as hormone levels fluctuate. Moreover, the dual effects are exhibited by androgen.

### Estrogen signalling

Sex-differences in stress response and reactivity [e.g., 337–339] can be explained by the fact that estrogen influences neurocognition, such as working memory, episodic memory, social memory, spatial memory, selective attention, and memory system bias [e.g., 115, 340–345]. Estrogen-facilitated hippocampal activity has been shown to exhibit a linear and inverted U-shaped dose–response effect in young women [e.g., 345]. Post-menopausal reduction of estradiol can be a risk factor for cognitive decline [e.g., 346]. However, estrogen plays a dual role and is either neuro-protective or neuro-harming in the stress response [339, 347; see also 348, 349].

We postulate that the estrogen effect is a function of the estrogen signalling type (nuclear or non-nuclear) × environment.

#### Nuclear signalling and acute stress

Like GRs, activated estrogen receptors (ERs) can affect memory and behavior through regulation of gene transcription. When activated by binding estradiol, ERs can translocate to a nucleus and connect to estrogen response elements on DNA. This is a nuclear type of ERs activation with the direct genomic effects [e.g., 115, 342]. In other words, ERs dirrectly regulate transcription of the genes with cAMP response elements (CRE) in their promoters (e.g., *bdnf* and CRF genes) and, in turn, activate or attenuate CREB function that enhances or impairs, respectively, memory formation [e.g., 350; Figs. 1 and 4].

Although elevated estradiol levels can promote rapid hippocampal CREB phosphorylation, during acute stress, the outcomes can be harmful [e.g., 351]. For example, the effect can be seen in the amplified stress response possibly due to the co-activation between ERs, GRs and CRH-1 receptor [e.g., 352], impaired fear extinction [e.g., 353], and PFC-mediated working memory [354]. We predict that estrogen-related cognitive vulnerability is associated with an altered Ca^2+^/MK IIα—CREB pathway. In fact, estrogen can rapidly hyperactivate Ca^2+^/MK IIα via nongenomic pathways in the hippocampus [e.g., 254, see also 255].

#### Non-nuclear signalling and chronic stress

Circulating estradiol can also bind to membrane-specific estrogen receptors (mERα and nERβ) and G protein-coupled ERs (GPERs). Activated mERs can trigger rapid (within seconds to 5 min) nongenomic mechanisms in the dorsal hippocampus that involve metabotropic glutamate receptor GluA1 (aka GluR1) interactions. Next, indirect genomic effect can be seen within hours or days due to subsequently modulated CREB phosphorylation and CRE-dependent gene expression via MAPK [e.g., 355–359]. Furthermore, ER signalling can influence NE signalling [e.g., 360; see also 361]. The mnemonic effect of ERs correlates with D_1_-receptor dopamine activity [e.g., 234] and depends on a photoperiod (melatonin effect) that mediates estradiol-induced aggression [362, 363; see also 339; Photoperiodic Plasticity section below].

Estrogen has been found to support cognitive resilience to chronic or repeated stress in females [e.g., 364–367]. For instance, in contrast to male rats, chronic restraint stress (2-h/day for a week) did not impair PFC functions in female rats due to ER-related protective effect seen in the preserved temporal order recognition memory and AMPA/NMDA receptors subunits surface expression (i.e., GluR1/2 and NR1/2A/2B) [367].

The mechanistic parallelism is observed in neurological diseases. Specifically, in a mouse model of Alzheimer's disease, GPER30 activation was found to have an ameliorative effect on object recognition memory [368] and selective activation of non-nuclear ERs normalized the mitochondrial apoptotic pathway via pathway-preferential estrogen (PaPE)-1 involved in MAPK and mTOR [e.g., 369]. In a mouse model of stroke, selective non-nuclear ERs stimulation with PaPE-1 also decreased stroke severity and neuroinflammation in the brain in female mice [370]. Furthermore, ERs signalling is implicated in microvascular mechanisms which serve both cerebro- and cardio-protection via mERα-mediated endothelial effects (i.e., rapid dilatation and repair acceleration) and mERβ- mediated synthesis of nitric oxide that play a hypotensive role [371–376; see also 358, 367, 377].

Hence, despite the neuro- and cardio-protective properties of estradiol, evidence indicates that high estrogen levels increase cognitive sensitivity to stress and affect disease risk. We thus hypothesize that in the context of an extreme, acute stress response, nuclear ERs act as transcription factors that enable stress effects related to activated Ca^2+^/MK IIα (i.e., higher estrogen levels are harmful). This may partly explain why the prevalence of PTSD is twice as high in women than men [e.g., 378–380]. We also hypothesize that in chronic stress, non-nuclear ERs regulate the translational status, via histone modifications, to favour neuroprotective effects via PaPE-1 activation (i.e., higher estrogen levels are beneficial). Accordingly, estradiol treatment of women that were exposed to interpersonal violence has been shown to attenuate negative effect of severe PTSD symptoms on fear habituation [e.g., 381]. Moreover, mERs are considered a novel treatment target for age-associated memory decline, stroke, and neurodegenerative diseases [e.g., 382, 383].

### Testosterone

Similar to estrogen, androgen displays dual effects. Specifically, testosterone can enable (1) hippocampal synapse formation and spatial memory or (2) inhibition of BDNF and reduction in astroglial density that affects synaptic plasticity in men. In contrast to estrogen, androgen is not associated with social memory but social recognition if a male conspecific [e.g., 384, for additional mnemonic effects, see also 385–391]. The animal models of stress conditions, such as conditioned fear and inhibitory avoidance tasks, show that androgen’s metabolite 3α-diol can bind to mERβ and enhance hippocampal memory [392, 393]. Androgen is also implicated in observed sex-differences in stress reactivity related to impulsive behavior in rats, with males preferring large and delayed rewards and females preferring small and immediate rewards [394]. Clinically, this androgen effect may help explain why women have been found to be more affected by the frequency of the exposure to stress, whereas men appear to be more affected by the severity of stress [395; see also 339, 396].

## Photoperiodic plasticity

*Summary of the concepts*: Altered circadian rhythm with shortened daylight can increase neurocognitive vulnerability to stress.

*Takeaway*: Pharmacotherapy aimed at melatonin and serotonin signalling has the potential to attenuate cognitive deterioration associated with altered photoperiodic plasticity.

Changes in photoperiod can modulate stress-induced cognitive and affective disorders, such as those associated with seasonal differences. For example, greater stress vulnerability was observed in a rat model during exposure to short days [e.g., 397]. The underlying mechanism appears to be that shortened photoperiod can alter hippocampal volume and dendritic morphology related to the synaptic plasticity (i.e., decreased apical CA1 spine density and increased basilar CA3 spine density), resulting in poor spatial learning and memory [e.g., 398–400]. This mechanism involves activation of the melanocortin-4 receptors that promote aversive memory formation via protein kinase A signalling [e.g., 220]. The melanocortin-4 receptors are highly expressed in the medial amygdala [401]. Moreover, their activation increases synaptic plasticity via the dendritic spine morphology and abundance of the AMPA- receptors [e.g., 220]. The melanocortin-4 receptors activation is linked to rapid anxiogenic and anorectic effects in response to emotional stress [e.g., 401]. Stress-induced anxiety and depression can be aggravated by the melanocortin-4 receptors’ agonists and mitigated by the receptors’ antagonists [e.g., 402]. At the same time, the evidence suggests that reduced melatonin signalling is implicated in PTSD, psychiatric disorders, and neurodegenerative diseases [e.g., 397, 403–405]. Specifically, in PTSD, melatonin treatment can mitigate PTSD-like behaviors (related to contextual fear memory) and restore cortisol levels [e.g., 403], as well as improve spatial cognitive impairment via genomic mechanisms that increase CREB protein and mRNA levels in the hippocampus [397]. Melatonin supplementation can also attenuate Alzheimer’s-like tau hyperphosphorylation and β-amyloid aggregation, slow down cognitive deterioration, and improve sleep and sundowning in Alzheimer’s disease [e.g., 405–410]. Furthermore, researchers have also demonstrated that melatonin effects relate to serotonin and dopamine co-signalling. For example, antidepressant treatment with agomelatine that displays a synergistic effect on melatonergic MT_1_/MT_2_ and serotonergic 5-HT_2C_ receptors (agonist and antagonist, respectively) reduces stress-induced glutamate release in the PFC [e.g., 411, 412] and likely reduces depressive symptoms via circadian rhythms restoration following resynchronized sleep–wake cycle [e.g., 413, 414]. At the same time, circadian rhythmicity is bidirectionally interconnected to the stress system [e.g., 415] and relates to dopamine signalling [e.g., 416].

## Psychoneuroimmunologic effects

*Summary of the concepts*: Psychological stress can trigger neuroimmune and proinflammatory responses that influence mood and cognitive decline. In addition, a compromised immune system increases brain vulnerability to stress and neuropsychiatric conditions.

*Takeaway*: Stress coping and neuroinflammation are related. Indeed, psychological interventions can improve stress resilience via immunologic/anti-inflammatory mechanisms.

In reviewing stress-induced neuronal pathophysiology, it is important to highlight that brain networks communicate with the immune system to monitor and respond to many kinds of threats, including social, physical, and psychological stressors. Psychological stressors can trigger anticipatory neuroimmune responses that reduce risk for injury and infection by upregulating inflammatory activity in the mere presence of social threat [e.g., 417–419]. Therefore, even a “painful” feeling such as shame, but not guilt—which is an understanding that our actions have harmed somebody else—can increase proinflammatory cytokine activity while not affecting cortisol levels in healthy adults [e.g., 420]. Consistent with this model, PTSD has been shown to be associated with immune dysregulation [e.g., 421, 422]. Vice versa, a compromised immune system increases brain vulnerability to stress. For example, after controlling for the effects of age, education, and depression/anxiety, in men with HIV, it has been shown that stressful life events were related to cognitive deficit, in contrast to men who were HIV-negative with the same level of stress perception [423; see also 424]. Stress-associated verbal memory deficits have been found to be larger for HIV-positive as compared to HIV-negative women [425].

Rat models of stress provide further insights into how stress coping and neuroinflammation interconnect. For example, exposure to brief social defeat induces circulating inflammatory cytokines and primes neuroinflammatory responses (with the engagement of the LC-NE system) to a subsequent defeat exposure. Moreover, social defeat enhances neuroinflammation in the central amygdala but reduces it in the dorsal raphe [426]. In a repeated resident-intruder stress model in rats, coping strategy affects psychosocial stress susceptibility associated with neuroinflammation measured by interleukin-1β: whereas passive coping was linked to greater inflammation, active coping and stress resistance was linked to lesser inflammation [427, see also 428, 429].

Furthermore, the LC activity enables scanning attention and the analysis of behavior while actions are “on hold so the challenge can first be inspected” [e.g., 1]. It has also been shown that escape responses [56] and subordinate behavior are promoted by CRF influence yet supressed by opioid influence [428]. Hence, we may say that whereas stress-associated hyperarousal enables passive coping and neuroinflammation, emotional downregulation can facilitate active coping and reduce proinflammatory responses. In fact, research has shown that emotion regulation is associated with inflammatory activity levels [e.g., 430], and adequate coping strategies that alter cognitive appraisals and emotional responses can improve health outcomes [e.g., 431]. Given that neuroinflammation is implicated in neurocognitive/psychiatric disorders and neurodegenerative diseases [e.g., 429, 432–435], this research highlights the potential for psychological interventions such as cognitive and dialectical behavior therapy to improve vulnerable brain function. Indeed, a meta-analysis showed that psychosocial interventions such as cognitive behavior therapy reliably improve immune system function for at least 6 months following treatment [436].

The active role in neuroinflammatory regulation belongs to the cerebral glia—that is, microglia and astrocytes—as well as the endotheliocytes and peripheral immunocytes. Here, “the primary immune surveillance and macrophage-like activities” are performed by microglia [e.g., 437; see also 438], where neuroinflammatory processes are mediated via the LC-NE signaling system [e.g., 439]. Additionally, stress-induced changes exhibit sex-differences in the corticolimbic microglia. Specifically, dendritic re-modeling is seen mostly in the basolateral amygdala in females, as compared to wider microglial cell activation in males, which involve the basolateral amygdala, dorsal hippocampus, medial PFC, and corticolimbic projections [440].

Evidence also suggests that chronic social stress induces genome-wide transcriptional shifts characterized by increased proinflammatory and reduced anti-viral skewing via β-adrenergic signaling [441, 442]; this resonates with the transcriptional changes evident in PTSD [443]. Particularly, this shift involves the upregulation of target genes for a proinflammatory immune response (e.g., NF-κB and AP-1) and a reciprocal downregulation of target genes for an anti-inflammatory response (e.g., GRs coding gene *NR3C1* and interferon response factors). This proinflammatory shift explains why chronic social stressor exposure is related to high levels of morbidity and mortality [441].

At the same time, single nucleotide polymorphisms in the promoters of proinflammatory immune response genes, such as *IL6*, influence DNA binding affinity that in turn affects the extent to which social threat-activated GATA1 transcription factor activity predicts longevity. In this context, individuals with GATA1-sensitive G allele have been found to have higher levels of DNA binding affinity and *IL6* gene expression—which is associated with earlier mortality—as compared to their counterparts with the GATA1-insensitive C allele [441, 444]. To complicate the matter, altered immune functioning/inflammatory responses (e.g., levels of IL-6, TNF-α, and C-reactive protein), as well as the polymorphisms in *IL6, IL1β*, *IL10*, and *TNFIα*, appear to contribute to a potential increased susceptibility to depression, cognitive impairment, and dementia/Alzheimer’s disease [e.g., 445–452]. Accordingly, modulating immunity appears to be a therapeutical target to support cognitive function in stress-related psychiatric conditions [e.g., 453].

## Conclusion and application

The Global Burden of Disease Study 2019 revealed that “mental disorders remained among the top ten leading causes of burden worldwide, with no evidence of global reduction in the burden since 1990” [454, p. 137]. Moreover, the authors wrote that “Mental health needs are high but responses are insufficient and inadequate. […] About one in eight people in the world live with a mental disorder” [455, p. iv]; “there remains much to be done to ensure all people achieve the highest standard of mental health and well-being. Action must be taken” [454, p. v]. Such conclusions expose a critical gap between the sizable magnitude of this public health crisis and our lackluster global effort to identify novel therapies that can successfully address the problem. To begin to address this issue, we reviewed research aimed at better understanding how stress affects the brain in a way that hampers integral aspects of mental health, including psychosocial well-being, cognitive functioning, and mental resilience.

**Fig. 7:**
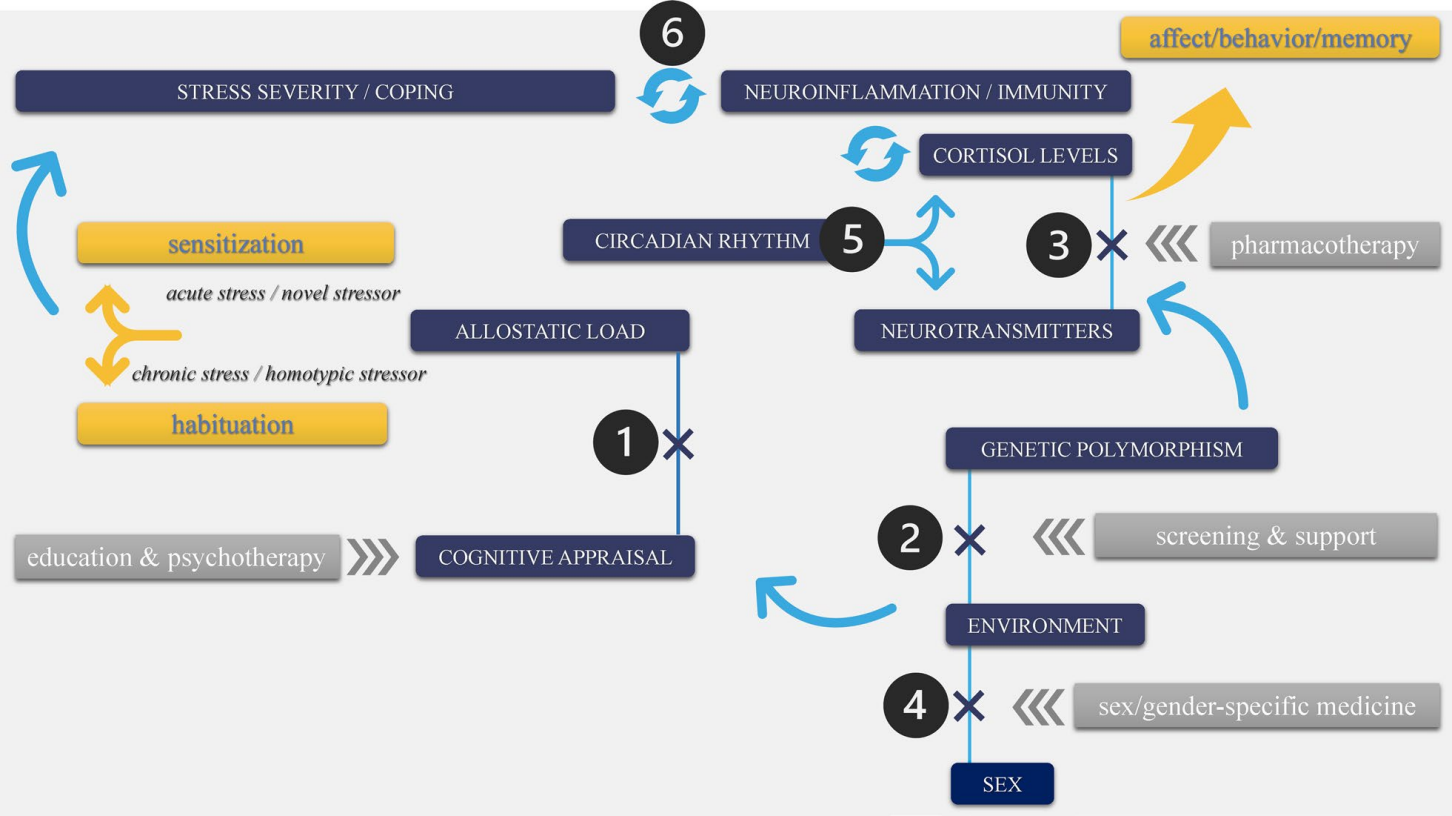
Determinants of Cognitive Resilience to Psychological Stress. Simplified and schematic model of biological processes linking psychological stress and cognition. Psychological stress can induce multiple neurobiological mechanisms related to cognitive decline and behavioral change. Principal determinants of stress resilience, which may support clinical interventions, are shown: (1) Psychobiology; (2) Epigenetics; (3) Neurotransmitters; (4) Sex hormones; (5) Circadian rhythm; and (6) Psychoneuroimmunology (see the Conclusion and Application section)

This research shows that psychological stress increases the risk for cognitive problems, and the development of serious neurological and psychiatric disorders [e.g., 3, 5–8, 456]. The exact mechanisms of stress-related cognitive deterioration and neuropsychiatric outcomes have yet to be clarified. To provide a fruitful path forward, we applied a multilevel approach that discerns shared cellular mechanisms underlying stress reactivity and neurocognitive processing (Fig. 7). In doing so, we elucidated the multilevel determinants of stress resilience, which may support clinical interventions. However, to realize the full potential of this work for addressing stress-related disease burden, these models will need to be translated into novel therapeutics that are safe, effective, widely accessible, and affordable.

### Level 1: Psychobiology

Stress reactivity is a function of a person’s cognitive appraisal and allostatic status; moreover, stress-induced habituation and sensitization are associated with harmful effects. Access to psychosocial education and services in community-based settings can enhance individual stress resilience. Psychotherapeutic interventions, such as cognitive behavior therapy, can help people cognitively reappraise their ability to deal with a stressor and, in turn, reduce their perceived stress severity and health risks [e.g., 1].

### Level 2: Epigenetics

The genetic polymorphism × sex × environment (e.g., level of social safety) interplay is associated with interindividual differences in stress-responses outcomes, such as morbidity risk and longevity [e.g., 441]. Proactive screening and proper support are necessary for individuals at particular risk of mental illness (e.g., genetic testing for physiological and behavioral traits, supporting marginalized groups, [e.g., 457]).

### Level 3: Neurotransmitters

Stress can affect multiple neurotransmitters related to neuronal plasticity and stress resilience. We hypothesize that the stress effects can be determined by the enzyme-protein dynamics. Specifically, anxiety and PTSD following acute and/or severe stress can be driven by altered Ca^2+^/MK IIα pathway, which is a fast nongenomic response with indirect epigenetic effect involving CREB mechanism (Fig. 5). We also hypothesize that depression and neurodegeneration following chronic stress can be driven by SNARE protein complex accumulation in synaptic membranes linked to excito-/proteo-toxicity and slow genomic effect involving ERK/MAPK (Fig. 6). Pharmacotherapy based on neurotransmitter signaling and administrated to block harmful effects of glucocorticoids can help stress resilience and neurocognitive functioning.

### Level 4: Sex hormones

Estrogen and androgen signalling influence memory and behavior, which is linked to stress resilience. We hypothesize that dual, neuro-protective or neuro-harming, role of estrogen effect is a function of the estrogen signalling type (nuclear or non-nuclear) × environment (exposure to acute or chronic stress). Advanced mental health care requires promoted sex/gender-specific medicine.

### Level 5: Circadian rhythm

Altered circadian rhythm with shortened daylight can increase neurocognitive vulnerability to stress. Addressing circadian dysrhythmia and associated alterations in melatonin and serotonin signalling can support brain functions.

### Level 6: Psychoneuroimmunology

There is a bidirectional association between stress coping and immunity. Interventions aimed at enhancing immunity and emotional intelligence may have a protective impact on neurocognitive resilience to stress.

Additional research is needed to test the hypotheses described. To this end, the present model provides a fruitful avenue for investigating aspects of cellular neuropathophysiology that can help advance our understanding of features of the stress responses that contribute to, and interplay with, psychiatric and neurological disorders. The outlined determinants of stress resilience can thus assist cross-disciplinary clinical and translational neuroscience to promote brain and mind health.

## Data Availability

Not applicable.

## Abbreviations

5HTTLPR: Serotonin transporter-linked promoter region
ACTH: Adrenocorticotropic hormone
BDNF: Brain-derived neurotrophic factor
Ca^2+^/MK: Ca^2+^/Calmodulin-dependent kinases
COMT: Catechol-*O*-methyltransferase
CRE: CAMP response elements (CRE)
CREB: CAMP-response element-binding protein
CRF: Corticotropin-releasing factor
CRH: Corticotropin-releasing hormone
ER: Estrogen receptor
ERK: Extracellular regulated kinase
GR: Glucocorticoid receptor
HPA: Hypothalamic–pituitary–adrenal
Hsp: Heat shock proteins
LC-NE: Locus coeruleus-norepinephrine
MAPK: Mitogen-activated protein kinase
MR: Mineralocorticoid receptor
PFC: Prefrontal cortex
PTSD: Post-traumatic stress disorder
SNARE: Soluble *N*-ethylmaleimide-sensitive factor attachment protein receptor
SPS: Single prolonged stress
TSST: Trier Social Stress Test
